# Impact of Melatonin as a Premedication Agent in Caesarean Section on Blood Loss and Postoperative Pain Level

**DOI:** 10.1155/2023/8102111

**Published:** 2023-12-12

**Authors:** Hussein Alkhfaji, Mohamed Kahloul, Talib Razaq M. Askar, Majid Fakhir Alhamaidah, Hussein Ali Hussein

**Affiliations:** ^1^Department of Anesthesia, College of Health and Medical Technology, Al-Ayen University, Nasiriyah, Iraq; ^2^Department of Anesthesia and Intensive Care, Faculty of Medicine Ibn El Jazzar, University of Sousse, Sousse, Tunisia; ^3^Department of Anesthesia and Intensive Care, Sahloul Teaching Hospital, University of Sousse, Faculty of Medicine of Sousse, Sousse, Tunisia; ^4^Thi Qar University Medical College, Nasiriyah, Iraq

## Abstract

**Background:**

Postpartum hemorrhage (PPH) is a serious postdelivery condition with a high incidence of morbidity and mortality for women who undergo childbirth with or without a caesarean section. Melatonin has been suggested to increase the contractility of myometrium and reduce the pain score postoperatively, therefore it is believed that the use of melatonin before surgery may decrease blood loss, reduce pain score, and decrease the need for postoperative opioids.

**Objectives:**

The main objectives of this study are focused on the investigation of melatonin as a premedication agent to reduce blood loss and decrease pain score postoperatively in patients undergoing cesarean section under spinal anesthesia.

**Methods:**

80 patients were scheduled for spinal anesthesia-based cesarean sections and randomly assigned to two groups, melatonin group (M) 40 patients and placebo group (P) 40 patients to receive either 10 mg of sublingual melatonin or a placebo of 90 minutes preoperatively. Hemoglobin levels were been measured preoperative and 12 hrs. Postoperatively, blood loss volume was calculated by measuring both the weight of used materials before and after the surgery and the volume sucked in the suction bottle after placental delivery. Postoperative visual pain score and analgesic requirements were used to evaluate pain levels.

**Results:**

Analyzed collected data showed a significant decrease in blood loss in the melatonin group in comparison with the placebo group as measured by the hemoglobin level. On the other hand, there is a significant decrease in pain score and analgesia requirement with the melatonin group compared to the placebo group.

**Conclusion:**

Melatonin is a promising premedication drug that has a significant impact on postpartum hemorrhage by reducing blood loss and pain levels of mothers who have undergone C-sections.

## 1. Introduction

The hormone melatonin, also known as N-acetyl-methoxytryptamine, has been found to have a significant impact on uterine physiology, as well as well-known effects on reproduction that are mediated through the central nervous system [1]. The notion is substantiated by the recent discovery of melatonin MT1 and MT2 receptor transcripts in the human myometrium [2]. In addition, it has been documented that levels of melatonin experience an increase in the maternal bloodstream, amniotic fluid, and urine of expectant mothers during the course of pregnancy, culminating in a zenith at full term [3]. Postpartum hemorrhage, or PPH, is a common disease marked by a maternal blood loss of 500 ml or more within 24 hours following delivery. According to estimates, this illness affects 6% of women who give birth [4]. The primary cause of maternal mortality globally is obstetric hemorrhage, which accounts for 27% of the total 295 × 10^3^ maternal deaths that occur annually. Postpartum hemorrhage is responsible for over two-thirds of these deaths with high incidence in Asian and African nations [5]. The administration of melatonin during a caesarean section is believed to have a significant impact on minimizing blood loss throughout the operation [6]. Vasoconstriction, which is characterized by the constriction of blood vessels and subsequent decrease in oxygenated blood flow to the targeted area, may be a potential mode of action for melatonin [7]. Melatonin has demonstrated potential advantages in acute pain scenarios, such as postoperative pain. The administration of melatonin either preoperatively or in the immediate postoperative period has been linked to a reduction in pain scores and a decrease in opioid requirements in certain studies [8]. The aim of this study is to investigate the impact of melatonin as a premedication agent in caesarean section on blood loss and pain levels.

## 2. Methods

After obtaining the institutional ethics committee's approval with code 37/2021 and patients' informed consent, this prospective randomized double-blind study was conducted in the operating theatre of caesarean sections in Bint Al-Huda Teaching Hospital in Nasiriyah, which is located in Dhi Qar Governorate, southern Iraq, during the period spanning August 2021 to January 2022. The sample size determination is based on changes in blood loss, considering the variations in blood loss. The effect size was found to be 0.45 with a standard deviation of 0.71. The minimum sample size was calculated to be 80 patients who were divided into two groups, each group consisting of 40 patients, in order to maintain a minimum power of 80% and a maximum type I error of 0.05 (the sample calculation was performed online at https://marne.u707.jussieu.fr/biostatgv/). All caesarean section patients agreed to take part in the experiment. The study's inclusion criteria encompassed individuals who fulfilled the subsequent requirements: age exceeding 18 years, categorized as ASA I or ASA II, pregnancy at a gestational age surpassing 37 weeks, unbroken membranes, scheduled surgical procedures, patient agreement and contentment with study participation, and women with a solitary pregnancy.

The exclusion criteria for the study encompass several factors, including ASA III or higher, which indicates severe systemic disease or a constant threat to life, documented drug allergy to melatonin or any other study medications, contraindications for spinal anesthesia such as spinal abnormalities or infections, inability of the patient to respond or demonstrate awareness to the questions rose, patient with chronic anemia (Hb) <8 g%, a history of mental or neurological diseases that could affect the patient's ability to participate or comprehend the study procedures, addiction to substances that could interfere with the study outcomes, disapproval or dissatisfaction expressed by the patient regarding their involvement in the study, presence of congenital malformations in the fetus detected during routine prenatal screening, inability to provide informed consent due to intellectual impairment or other factors, and significant heart disease that could pose additional risks during the surgical procedure. Eighty patients who had been scheduled for cesarean section under spinal anesthesia were enrolled in the study. We randomly assigned 40 patients to each group, melatonin (M), and placebo (P). An epidemiologist created a random sequence for administering study substances (melatonin and placebo) in a 1 : 1 ratio, delivered by a nonparticipant in the clinical study. Melatonin and placebo, visually identical and prepared by a specialist pharmacist, were individually packaged. The researcher and participants could not discern the administered drug. A sublingual dose of 10 mg was given to patients 90 minutes before the operation and at night preoperative. We measured hemoglobin levels before and 12 hours after surgery, mean weight of materials used during surgery, and the amount of blood suction. Visual pain score and analgesic administration were used to measure the level of pain, and all patients had standard monitoring (electrocardiogram, noninvasive blood pressure, and pulse oximetry). Before subarachnoid block administration, all subjects were administered a lactated Ringer's solution intravenous preload at a rate of 5–7 ml/kg. Following the implementation of an aseptic technique, a Quincke needle with a gauge of 25 was introduced intrathecal through a midline approach into the L4-5 interspace; while the patient was in a seated position, Marcaine 10.5–14 mg (1.4–2 ml) was used. The procedure was performed by the same resident who was not cognizant of the task assigned. Following the delivery of the neonate, oxytocin was administered intravenously through a 15-minute infusion of oxytocin dissolved in 500 ml of lactated Ringer's solution; the dose was determined by the surgeon who deemed uterine tone to be insufficient. Hemoglobin levels were assessed prior to and 12 hours subsequent to the surgical procedure. In addition to the pre- and postsurgery hemoglobin level changes, blood loss was assessed by using two methods: (a) the weight difference of materials used before and after surgery and (b) the volume of blood collected in the suction bottle following placental delivery, both measured in milliliters (ml). A standardized protocol was employed to administer uniform spinal anesthesia to all patients during the surgical procedures. After undergoing surgical interventions, patients were admitted to the ward and a patient-controlled analgesia (PCA) pump was employed to manage pain for all patients. The internal composition of the PCA pump consists of 0.25% mg of morphine added to a normal saline solution. The adjustment parameters of the PCA pump comprised of a bolus size measuring 0.5 cubic centimeters and a lockout interval of 15 minutes, which stands for 0.2 mg/kg with the maximum dose not exceeding 15 mg. Chi-square analysis is a statistic which is commonly used for testing relationships between categorical variables [9]. The null hypothesis of the chi-square test is that no relationship exists between the categorical variables in the population; they are independent. Student's t T-test was used to assess the statistical significance of the difference between the two study group means. A paired *T*-test was used to assess the statistical significance of the difference between two periods; the Mann–Whitney Test (*U* test) was used to assess the statistical significance of the difference of a nonparametric variable between two study groups, and ANOVA with repeated measures test was used to assess the statistical significance of the difference between more than two period parametric variables. The collected data were revised, coded, and tabulated using the Statistical Package for Social Science (IBM Corp., released 2017, IBM SPSS Statistics for Windows, Version 25.0, Armonk, NY). Data were presented and suitable analysis was performed according to the type of data obtained for each parameter.

## 3. Results

**Figure 1:**
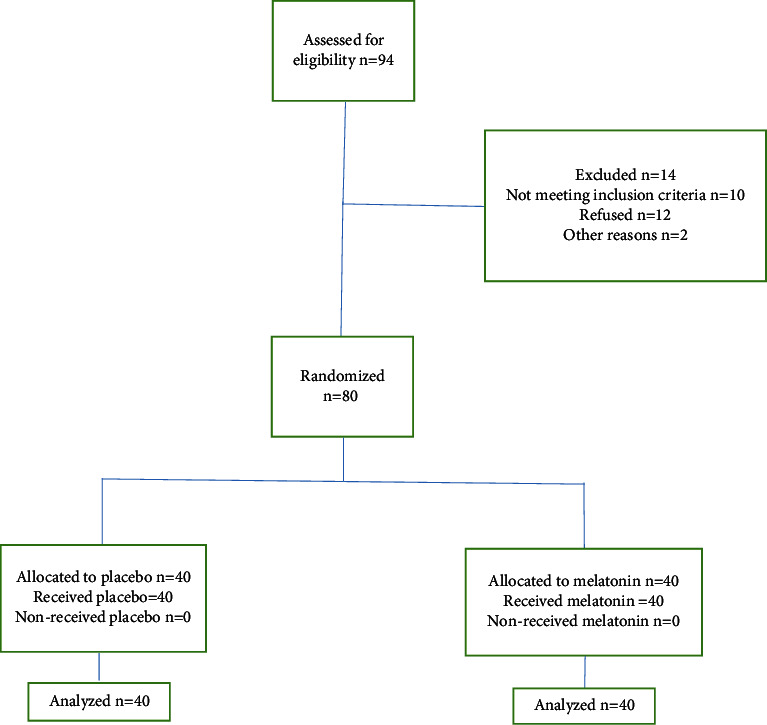
CONSORT flow diagram.

The research encompassed a cohort of 80 participants who were allocated randomly to two distinct groups according to the CONSORT flow diagram (Figure 1). No significant statistical differences were observed between the two groups with regard to their sociodemographic characteristics (Table 1). There were no statistically significant differences observed in the mean age, weight, height, and BMI between the study groups, with *p* values of 0.378, 0.144, 0.062, and 0.726, respectively.

The research conducted a comparative analysis on the impact of melatonin and placebo on individuals undergoing preoperative and postoperative interventions. The findings of the study suggested that individuals who were administered melatonin exhibited elevated levels of Hb in comparison to those who were given a placebo. The observed discrepancy was determined to possess statistical significance, as evidenced by a *p* value of less than 0.05. Furthermore, the research exhibited a reduction in hemoglobin concentrations in the postoperative phase among individuals who received either melatonin or a placebo. Comparing melatonin to a placebo in relation to hemoglobin levels. The study's results indicated that there was a significant statistical difference (*p* < 0.05) in the amount of blood loss between the two groups that were being examined. The results indicate that the mean blood loss value was significantly greater in the placebo group (*M* = 448.38, SD = 103.82) as compared to the melatonin group (*M* = 300.38, SD = 83.46). Comparison between melatonin and placebo regarding blood loss is shown in Table 2.

The present study conducted an analysis of pain scores to compare the severity of pain between the two groups as shown in Table 3. The results revealed that the median pain score was significantly higher in the placebo group than in the melatonin group during cannula, spinal needle, and after 30 mins, 2 hours, and 3 hours postoperatively (*p* < 0.05).

With regards to the administration of opioids postsurgery, a study was conducted comparing the effects of melatonin and placebo. The results showed that, after one hour, a significantly higher percentage of patients who received a placebo (17.5%) were administered opioids compared to those who received melatonin (5%). The observed difference did not reach statistical significance, as indicated by a *p* value of 0.05 or greater. Following a two-hour period, it was observed that 20% of patients who were administered placebo received opioid treatment, while only 10% of patients who were administered melatonin received the same treatment. The observed difference did not reach statistical significance, as indicated by a *p* value of 0.05 or greater. Following a three-hour period, it was observed that 57.5% of patients who were administered a placebo received opioid administration, whereas only 27.5% of patients who received melatonin were subjected to the same treatment. The observed distinction was deemed statistically significant at a level of *P* < 0.05 (Table 4).

## 4. Discussion

In recent times, melatonin has garnered the interest of scholars owing to its recently uncovered characteristics. Melatonin has been recognized for its natural hormone secretion by the pineal gland [10]. Furthermore, it has been found to possess significant anti-inflammatory properties [11] and has been utilized as a means to reduce blood loss during surgical procedures, particularly during cesarean delivery [3]. “Postpartum hemorrhage” (PPH) refers to excessive bleeding following childbirth, typically defined as blood loss of 1000 mL or more after a cesarean delivery [12]. It is a serious complication that can lead to maternal morbidity and mortality if not promptly and effectively managed. PPH is a prevalent complication that is often associated with cesarean deliveries, characterized by excessive bleeding following childbirth [13]. PPH can arise from multiple etiologies, such as inadequate uterine contraction, placental anomalies, or surgical mishaps [14]. The timely identification and effective handling of postpartum hemorrhage (PPH) are imperative in order to avert subsequent complications. Our study findings indicated the significant effect of melatonin in decreasing the amount of blood loss during C-section indicated by the hemoglobin level and the gauze weight. These findings are consistent with the study of Khezri et al. [3] who proved the effectiveness of melatonin as administration of 6 mg of melatonin via sublingual route as a premedication drug in patients undergoing cesarean section with spinal anesthesia has been found to result in a statistically significant reduction in the amount of blood loss following lower segment cesarean section. However, the clinical significance of this finding remains uncertain. The potential benefits of melatonin as an anxiolytic and uterotonic agent in obstetric settings during spinal anesthesia are noteworthy [15]. Given the elevated incidence of anemia in expectant mothers, a modest decrease in postpartum hemorrhage could hold clinical significance and alleviate patient discomfort [16]. Melatonin has a synergistic effect on the contractility of human myometrial smooth muscle cells induced by oxytocin through the activation of MT2R. This activation leads to an increase in the phosphorylation of the myosin light chain protein, which is dependent on protein kinase C [17]. The expression of MT2R was significantly higher in samples obtained from pregnant women who had initiated the process of labor than in pregnant women who were not in labor and matched accordingly. The expression of connexin 43, a gap junction protein, was upregulated by MEL. The results of *in vitro* dye spread assays indicate that the cells treated with MEL exhibited a significant enhancement in intercellular coupling. The upregulation of connexin mRNA and intercellular communication was observed to be facilitated by MT2R through a mechanism that is dependent on protein kinase C [18, 19]. Melatonin exhibits anti-inflammatory properties that potentially contribute to its analgesic effects. The perception of pain is influenced by inflammation, and the potential reduction of inflammation by melatonin may have analgesic effects [20, 21]. The modulation of pain signaling is a complex process that involves the interaction of various neurotransmitter systems responsible for pain regulation [22]. Our study's finding proved that melatonin had a significant effect on decreasing pain after C-section which is consistent with many other studies such as the study of Posa et al. [23], which revealed that MLT exhibits analgesic properties in various animal models of chronic, acute, inflammatory, and neuropathic pain. It is noteworthy that these effects are modulated by MT2 receptors, as evidenced by their inhibition by selective MT2 antagonists. The analgesic effects of two selective MT2 receptor partial agonists, UCM924 and UCM765, have been observed to be more potent than MLT in various pain paradigms, indicating the role of MT2 receptors in pain modulation. Studies have shown that melatonin exhibits effectiveness as both an analgesic and anxiolytic agent in both animal and human subjects [24]. There exists a proposition that the regulation of pain by melatonin is facilitated through the involvement of membrane receptors, nuclear receptors, and simple diffusion. Melatonin exhibits promising analgesic properties with minimal negative effects, indicating its potential as a pain-relieving agent [25]. The precise mechanisms through which melatonin may have contributed to the observed outcomes of decreased opioid administration in the study remain incompletely comprehended and necessitate additional inquiry. There exist various potential rationales for the mechanism through which melatonin may exert such an impact [26]. Melatonin has been observed to exhibit analgesic properties in both preclinical and clinical investigations [27]. The modulation of pain perception and the alleviation of pain can be achieved through interactions with particular receptors and neurotransmitters that are involved in pain signaling pathways [28]. Melatonin demonstrates anti-inflammatory properties by suppressing the secretion of proinflammatory cytokines and diminishing oxidative stress. It is widely acknowledged that pain sensitization can be attributed to inflammation [29]. Melatonin, by virtue of its ability to reduce inflammation, may serve as an indirect means of mitigating pain [30]. The central nervous system's opioid receptors can be modulated by melatonin, as evidenced by research [31]. The influence of a certain factor has the potential to affect the binding and activity of opioids, which may result in the enhancement of their analgesic effects and a decrease in the requirement for exogenous opioids [32].

Melatonin has the potential to mitigate pain and decrease the necessity for opioid medications by safeguarding neurons against harm [33]. One of the strengths of this study is its approach in using placebo and melatonin in a randomized control trial. In essence, this methodology guarantees the accuracy and consistency of the research outcomes and facilitates the utilization of empirical data for informed decision-making in the realm of clinical practice.

## 5. Conclusion

The study's results suggest that melatonin may have potential as a premedication agent for caesarean section procedures. The data indicate that melatonin may have an extended analgesic impact, as evidenced by the decrease in opioid administration observed after a three-hour period. Further research endeavors should aim to investigate the underlying mechanisms that give rise to this phenomenon and examine the most effective utilization of melatonin in the context of pain management interventions. The incorporation of melatonin as a supplementary treatment has the potential to enhance postoperative results and enhance patient contentment within this surgical context.

## Figures and Tables

**Table 1:** Comparison between melatonin and placebo groups regarding demographic data.

	Melatonin, *n* = 40	Placebo, *n* = 40	Test (*p*)
*Age (years)*			
Mean ± SD.	28.85 ± 7.46	30.20 ± 6.08	*t* = 0.887, *p* = 0.378
Median (range)	28.50 (20.0–49.0)	30.0 (18.0–42.0)	

*Weight (kg)*			
Mean ± SD.	77.60 ± 10.90	74.48 ± 7.76	*t* = 1.477, *p* = 0.144
Median (range)	76.0 (60.0–120.0)	73.50 (59.0–95.0)	

*Height (cm)*			
Mean ± SD.	165.03 ± 7.91	162.30 ± 4.43	*t* = 1.902, *p* = 0.062
Median (range)	165.0 (150.0–185.0)	162.0 (155.0–173.0)	

*BMI (kg/m* ^*2*^)			
Mean ± SD.	28.50 ± 3.37	28.27 ± 2.64	*t* = 0.351, *p* = 0.726
Median (range)	28.44 (20.76−7.04)	27.75 (22.65–34.34)	

SD, standard deviation; range, min–max; *t*, Student's *t*-test; *P* value compared between melatonin and placebo.

**Table 2:** Comparison of mean and Sd. of hemoglobin and blood loss.

Mean and standard diffusion of Hb according to drugs preoperative and postoperative				
Drugs	Preoperative mean and Sd.	Postoperative mean and Sd.	*P* value	Blood loss (ml)
Melatonin	10.79 ± 1.24	10.08 ± 1.19	0.012^*∗*^	300.38 ± 83.46
Placebo	10.13 ± 1.30	9.07 ± 1.14	<0.001^*∗∗*^	448.38 ± 103.82
*P* value				<0.001^*∗∗*^

SD, standard deviation; range, min–max; *t*1, Student's *t*-test; *t*2, paired *t*-test; *P*1 value compared between melatonin and placebo; *P*2 compared preoperative and postoperative; ^*∗*^significant when *p* value <0.05.

**Table 3:** Comparison of visual pain scores.

Pain score	Melatonin, *n* = 40	Placebo, *n* = 40	Test (*p*)
*During cannula*			
Median (range)	2.0 (1.0–5.0)	3.0 (1.0–8.0)	*U* = 1276.0, *p* < 0.001^*∗*^
IQR (25–75)	2.0–3.0	3.0–5.0	

*During spinal needle*			
Median (range)	3.0 (2.0–5.0)	5.50 (3.0–10.0)	*U* = 1372.0, *p* < 0.001^*∗*^
IQR (25–75)	2.0–4.0	3.50–7.0	

*Post 30 min*			
Median (range)	1.0 (0.0–2.0)	0.0 (0.0–4.0)	*U* = 428.0, *p* < 0.001^*∗*^
IQR (25–75)	0.0–1.0	0.0–0.0	

*Post 1 h*			
Median (range)	0.0 (0.0–2.0)	1.0 (0.0–2.0)	*U* = 978.0, *p* = 0.063
IQR (25–75)	0.0–1.0	0.0–2.0	

*Post 2 h*			
Median (range)	1.0 (0.0–4.0)	2.0 (1.0–3.0)	*U* = 1000.0, *p* = 0.036^*∗*^
IQR (25–75)	1.0–2.0	1.0–2.0	

*Post 3 h*			
Median (range)	2.0 (1.0–4.0)	4.0 (1.0–6.0)	*U* = 1280.5, *p* < 0.001^*∗*^
IQR (25–75)	2.0–3.0	3.0–4.0	

Range, min–max; *U*, Mann–Whitney; *P* value compared between melatonin and placebo; ^*∗*^significant when *p* value <0.05.

**Table 4:** Comparison of postoperative opioid administration.

Postoperative opioid administration	Melatonin, *n* = 40		Placebo, *n* = 40		Test (*p*)
	No.	%	No.	%	
*After 1 hour*					
No	38	95.0	33	82.5	*x* ^2^ = 3.130, Fisher exact *p* = 0.154
Yes	2	5.0	7	17.5	

*After 2 hours*					
No	36	90.0	32	80.0	*x* ^2^ = 1.569, *p* = 0.210
Yes	4	10.0	8	20.0	

After *3 hours*					
No	29	72.5	17	42.5	*x* ^2^ = 7.366, *p* = 0.007^*∗*^
Yes	11	27.5	23	57.5	

*x* ^2^, chi-square test; *P* value compared between melatonin and placebo; ^*∗*^significant when *p* value <0.05.

## Data Availability

The datasets used to support the findings of this study are available from the corresponding author on reasonable request.
